# Identification of hemostatic genes expressed in human and rat leg muscles and a novel gene (LPP1/PAP2A) suppressed during prolonged physical inactivity (sitting)

**DOI:** 10.1186/1476-511X-11-137

**Published:** 2012-10-12

**Authors:** Theodore W Zderic, Marc T Hamilton

**Affiliations:** 1 Inactivity Physiology Department, Pennington Biomedical Research Center, LA, Baton Rouge, 70808, USA

**Keywords:** Lysophospholipid, Lysophosphatidic acid, Physical activity, Hindlimb unloading, Sedentary

## Abstract

**Background:**

Partly because of functional genomics, there has been a major paradigm shift from solely thinking of skeletal muscle as contractile machinery to an understanding that it can have roles in paracrine and endocrine functions. Physical inactivity is an established risk factor for some blood clotting disorders. The effects of inactivity during sitting are most alarming when a person develops the enigmatic condition in the legs called deep venous thrombosis (DVT) or “coach syndrome,” caused in part by muscular inactivity. The goal of this study was to determine if skeletal muscle expresses genes with roles in hemostasis and if their expression level was responsive to muscular inactivity such as occurs in prolonged sitting.

**Methods:**

Microarray analyses were performed on skeletal muscle samples from rats and humans to identify genes associated with hemostatic function that were significantly expressed above background based on multiple probe sets with perfect and mismatch sequences. Furthermore, we determined if any of these genes were responsive to models of physical inactivity. Multiple criteria were used to determine differential expression including significant expression above background, fold change, and non-parametric statistical tests.

**Results:**

These studies demonstrate skeletal muscle tissue expresses at least 17 genes involved in hemostasis. These include the fibrinolytic factors tetranectin, annexin A2, and tPA; the anti-coagulant factors TFPI, protein C receptor, PAF acetylhydrolase; coagulation factors, and genes necessary for the posttranslational modification of these coagulation factors such as vitamin K epoxide reductase. Of special interest, lipid phosphate phosphatase-1 (LPP1/PAP2A), a key gene for degrading prothrombotic and proinflammatory lysophospholipids, was suppressed locally in muscle tissue within hours after sitting in humans; this was also observed after acute and chronic physical inactivity conditions in rats, and exercise was relatively ineffective at counteracting this effect in both species.

**Conclusions:**

These findings suggest that skeletal muscle may play an important role in hemostasis and that muscular inactivity may contribute to hemostatic disorders not only because of the slowing of blood flow per se, but also potentially because of the contribution from genes expressed locally in muscles, such as LPP1.

## Introduction

Low muscular activity has been associated with cardiovascular diseases [1,2]. In contrast to the more established literature on the effects of formal types of exercise, there is an emerging interest in the underlying molecular responses to physical inactivity and the more common non-exercise activities such as standing and low-intensity physical activity [3]. Deep venous thrombosis (DVT) can occur from an acute lack of normal ambulatory activity [4-6], and we are unaware of any evidence that it can be prevented by exercise [7,8]. For example, it was first reported over 60 years ago that sitting in London air raid shelters was associated with a 6-fold increased incidence of DVT [6]. Then, Homans noted in 1954 that the “spontaneous thrombosis which almost exclusively occurs in the legs” is due to “a state imposed by airplane flights, automobile trips, and even attendance at the theater” [4]. There was a report in that same decade of a patient with “thrombosis occurring as a result of prolonged sitting while the patient was watching television” [5] and the insidious risks of typical amounts of sitting or other sedentary behavior has continually been observed [9,10]. Currently, there are ∼2 million cases of DVT reported each year in the United States, leading to 250,000 deaths due largely to pulmonary embolism [11].

What distinguishes DVT from other forms of thrombosis is that it occurs deep in the muscle of the legs. Because the hypercoagulable state develops locally within the legs, there has been a long held supposition that dysregulation of cellular processes locally in the leg muscle tissue may somehow contribute to the risk of DVT in the legs. Support for this proposition would be buttressed by the detection of significant hemostatic gene expression (mRNA) in human skeletal muscle. The first inkling for this came from a recent report that there is a pool of tPA protein (tissue plasminogen activator) localized within the skeletal muscle tissue [12], consistent with the muscle producing tPA via local gene expression, but not ruling out the possibility that the protein was delivered to muscle from the circulation. In addition, a more recent study reported that skeletal muscle expressed not only tPA but also 3 additional hemostasis related genes (CTAP III, tetranectin, and PAI-1) in overweight men with metabolic syndrome [13]. Giving a further hint of a role for skeletal muscle in hemostasis, the expression of these genes was altered by 9 months of exercise training to result in a pattern that would be consistent with less risk of thrombosis [13]. As little is known about the role of skeletal muscle in hemostasis at this point, determining if skeletal muscle expresses a number of hemostasis related genes may open the door toward a new insight for the role of this tissue in hemostasis. Therefore, the first aim of this study was to comprehensively examine what hemostasis genes are expressed in skeletal muscle through the use of microarray technology. Furthermore, we sought to determine if any of these expressed genes would be altered by commonly used models of reduced contractile activity in the legs (hindlimb unloading in rats and sitting in humans). A translational study of rodent and human skeletal muscle was performed given the eventual need for physiological studies inducing thrombosis in experimental animal models while manipulating expression of select hemostatic genes. The identification of a hemostatic gene(s) impacted by prolonged inactivity like sitting may be important to investigate as a candidate for a role in the pathogenesis of DVT.

## Methodology

### Rat experiments

Animal procedures were approved by an Institutional Animal Care and Use Committee and performed in respect of the American Physiology Society principles for research on animals. Female Sprague Dawley rats (Harlan) weighing approximately 200 g were housed individually (450 cm x 241 cm) in a temperature- and light-controlled environment (12:12 hrs light–dark cycle). The soleus muscle (deepest skeletal muscle in the rat hindlimb) was obtained under anesthesia (16.2 mg ketamine, 0.66 mg xylazine, 1.05 mg acepromazine) from all rats and frozen in liquid nitrogen after the completion of the following described physical activity/inactivity treatments.

Altogether, 89 rats were used in these studies. In the first set of experiments, the effect of the acute (12 hrs) removal of normal standing/ambulatory activity on skeletal muscle gene expression was tested in rats. Rats with normal standing and ambulatory cage activity and spontaneous movements who never exercised served as controls for these acute experiments (n = 24). Forty-nine rats were restricted from standing on the hindlimbs for 12 hrs (7 PM to 7 AM, dark cycle) by wrapping 1.5 cm of the tail with adhesive tape connected to a fishing lure swivel tied to an overhanging metal rod (hindlimb unloading). This specific hindlimb unloading model and effects on muscle have been described previously [14]. The hindlimbs were elevated just enough to prevent the feet from touching the floor. All rats were acclimatized to the treatment of hindlimb unloading for at least 2 days before the experiments (1 hr/day). In order to test if exercise would quickly reverse the effects of inactivity, 20 of the inactive rats were exercised on a treadmill (8 m/min) immediately following 12 hours of not standing (hindlimb unloading). They exercised on a treadmill for an accumulated duration of two hours (4 x 30 minutes) over a 4 hour period of time (with allowance for spontaneous ambulation and standing in the cage the rest of the time). Food was withheld during hindlimb unloading and treadmill exercise.

In the chronic study (11 days), we sought to determine the impact of reduced muscle use per se as in the acute study, without the potentially extreme (up to 40%) muscle atrophy and associated remodeling caused by 1–2 weeks of continuous muscle disuse in rats [15]. To successfully accomplish this, 8 rats underwent the hindlimb unloading procedure for ∼10 hrs/day for 11 days, and then were euthanized 3 hours into the dark cycle. We previously reported that this completely prevents the muscle atrophy associated with muscle inactivity [14]. Eight control rats were euthanized at the same time of day in order to obtain the soleus muscles.

### Human experiments

All subjects volunteered to take part in this study after being informed of potential risks associated with participation and providing written consent. This study was approved by an institutional Internal Review Board. As in the rats, the effect of ∼12 hrs of muscle inactivity in the legs was studied. The primary study of inactivity was performed in three healthy adult males (31 ± 6 years, 69 ± 5 kg, 23.1 ± 0.9 kg/m^2^; mean ± SD) who exercised habitually (two subjects bicycled and one ran 3–4 days/week). Additionally, a more extensive case study was performed in a healthy 25-year old male as described in more detail below. On the standing/ambulatory control trial, subjects performed normal intermittent standing/ambulation restricted to light intensity activities. During the sitting trial, subjects worked on computers, watched videos, read, used the telephone, talked with others, and performed other sedentary activities for 12 hours. Subjects stood upright momentarily to use the toilet and were not sitting in an awkward position that might cause abnormal mechanical occlusion, so as to focus on the underlying singular effects of inactivity on gene expression. Approximately 100 mg was biopsied from deep within the vastus lateralis (6–9 cm in depth) after 12 hours of either sitting or after 12 hours of standing/ambulatory activity. In order to control for diet and activity, subject diets were replicated for the two days preceding each trial and physical activity was well controlled during the two days before each trial by disallowing any form of exercise. Furthermore, all subjects consumed identical dinners the night before the experimental trials and the same breakfast the morning of each experimental trial and fasted for 12 hours before all biopsy samples. Lastly, a case study was performed on one subject (66 kg, 21 kg/m^2^) during 15 days of wheelchair use. He was recreationally active (bicycling) and was estimated to have a VO_2max_ of 51 ml/kg/min from the heart rate responses to an incremental cycling ergometer test performed before he was subject to the physical inactivity treatments. One of his legs remained inactive during this time, while one leg was exercised daily by a combination of exercises without adding back the natural standing and other low-intensity physical activity. He performed two one-legged cycling bouts a day with one being a continuous 30 minute bout at 68 watts eliciting a rating of perceived exertion (RPE) of 12 (Borg scale) and another 30 minute session that included four 5-minute intervals at 108 watts eliciting an RPE of 15 (hard). He also performed unilateral resistance exercises at an intensity in which 6 sets of 20 repetitions could be completed with voluntary fatigue. Because of the potential concern for heterogeneity between biopsies within the same persons, we obtained duplicate biopsies (one in each leg from approximately the same area) to ascertain variability, and the mean inter-biopsy coefficient of variation for LPP1 gene expression was 6.5% (n = 3 duplicate biopsies).

### RNA processing

Muscle was homogenized on ice in TRIzol Reagent (GIBCO BRL) for three pulses of 15 seconds each, and total RNA was isolated. For the acute physical inactivity rat study, 10 mg muscle samples were pooled from 8–10 animals to minimize the impact of individual variability [16]. From each rat RNA pool or human biopsy, 18 μg of total RNA was converted into double-stranded cDNA using the SuperScript Choice system (GIBCO BRL) with an oligo-dT24 primer containing the T7 RNA polymerase promoter (Genset). Double-stranded cDNA was purified by phenol/chloroform extraction and precipitated. In vitro transcription was subsequently performed using an ENZO BioArray RNA transcript labelling kit (ENZO). Biotin-labeled cRNA was purified by an RNeasy kit (Qiagen) and fragmented.

There was a total of 3 pools of RNA from the control rats, 3 pools of RNA from the 12 hr inactive rats, and 2 pools of RNA from the treadmill exercised rats (8–10 rats per pool). For the chronic rat study, RNA processing yielded 2 pools of RNA from the control rats and 2 pools of RNA from the 11-day inactive rats (4 rats per pool).

### Microarray processing

Ten μg of fragmented biotinylated cRNA was hybridized for 16 hours on either the Affymetric human U133(A and B) or the rat U34A microarrays. Each microarray was washed and stained in the Affymetrix Fluidics Station 400 using the manufacturer’s instructions and reagents. This involved removal of non-hybridized material followed by incubation with streptavidin-phycoerythrin (SAPE) to detect hybridized cRNA. The signal intensity was amplified by a second staining with biotin-labeled anti-streptavidin antibody followed by SAPE staining. Fluorescent images were read before and after amplification using a Hewlett-Packard G2500A Gene Array Scanner.

### PCR analysis

Aliquots (2 μg) of total RNA from each biopsy were reverse transcribed with Superscript II RNase H Reverse Transcriptase (Invitrogen). PCR was then performed in a thermocycler (Mastercycler, Eppendorf) using Platinum Taq DNA Polymerase (Invitrogen). The LPP1 sense primer was GGT CTT GTT GCC GTA TCC AT and the anti-sense primer was TCC CAG TTG TTG GTG TTT CA (214 bp). GAPDH human primer sets (Clontech) were used to normalize data. Samples were loaded into a 3% agarose gel and dsDNA was visualized with Sybr Green I nucleic acid gel stain (Molecular Probes). Bands were quantified with densitometry using the STORM 860 (Amersham) scanner and ImageQuant software (Amersham).

### Statistical analyses

#### Microarray

Microarray images were analyzed using statistically based Affymetrix Microarray Suite 5.0 software [17]. In brief, each interrogated transcript was represented by 11 probe pairs for the human arrays and 16–20 probe pairs for the rat arrays. Each probe pair consists of a perfect match sequence (PM) that is complementary to the cRNA target and a mismatch sequence (MM) that includes a change of a single base critical for hybridization. Bacterial sequences were also included on the arrays as external controls for hybridization. Complete transcription and hybridization were validated using Affymetrix's recommended criteria based on the bacterial controls and several housekeeping genes. Published statistical algorithms [17] were used to determine whether or not a transcript was detectable in a given sample. Comparison of the hybridization signals from the PM and MM probes was performed with a one-sided Wilcoxon sign rank test by the Affymetrix software in order to determine if a gene was significantly expressed at a p-value below the false discovery rate (FDR) threshold [18]. The Wilcoxon non-parametric test was chosen because it is robust, insensitive to outliers, and does not need a normal data distribution [17]. Using the Affymetrix Net Affyx publicly available website (using terms such as clotting, coagulation, fibrinolysis, and hemostasis) and PubMed, we searched for the expression of genes related to hemostasis (Tables 1 and 2) and also ultimately lysophospholipid metabolism (Table 3) after identifying that the expression of lipid phosphate phosphatase 1 (LPP1) was affected by inactivity (see below).

**Table 1:** Hemostatic gene expression in human skeletal muscle tissue

**GENE**	**Access #**	**Expression**	**Frequency**	**P-value**	**Function**
**COAGULANT FUNCTIONS**					
**Coagulation factor VIII, procoagulant component**	**NM_000132.2**	**Strong detection**	**8/8**	**0.00024**	**Intrinsic pathway of blood coagulation**
**Coagulation factor VII**	**NM_000131.2**	**Strong detection**	**8/8**	**0.0016**	**Activates the coagulation cascade by converting factor IX to factor IXa factor X to factor Xa coagulation factor VII**
**Vitamin K epoxide reductase complex, subunit 1-like 1**	**NM_173517**	**Strong detection**	**8/8**	**0.0016**	**Paralog of vitamin K epoxide reductase complex, subunit 1**
**Coagulation factor VIII-associated (intronic transcript)**	**NM_012151.2**	**Strong detection**	**8/8**	**0.0020**	**unknown**
**von Willebrand factor (VWF)**	**NM_000552.2**	**Strong detection**	**8/8**	**0.005**	**Serves as a bridge between platelet glycoprotein GPIb/IX and collagen**
Vitamin K epoxide reductase complex, subunit 1	NM_024006	Moderate detection	8/8	0.014	Required for the carboxylation of glutamic acid residues in some blood-clotting proteins
Gamma-glutamyl carboxylase	BE326952	Moderate detection	7/8	0.024	Carboxylation of coagulation factors VII
			4/8	0.063	
Glycoprotein Ib (platelet), alpha polypeptide	NM_000173	Moderate detection	4/8	0.051	Binding of the GP Ib-IX-V complex to VWF facilitates initial platelet adhesion to vascular subendothelium after vascular injury
Coagulation factor XIII, A1 polypeptide	NM_000129.2	Moderate detection	3/8	0.068	Last zymogen to become activated in the blood coagulation cascade
Thrombin receptor	NM_001992.2	Moderate detection	4/8	0.071	Activated on surface of activated platelets by prothrombinase complex
Integrin, beta 3	NM_000212	Moderate detection	3/8	0.076	Promotion of the generation of thrombin by localizing prothrombin to cellular surfaces
			3/8	0.081	
**ANTI-COAGULANT FUNCTIONS**					
**Platelet-activating factor acetylhydrolase, isoform Ib, alpha subunit**	**NM_000430.2**	**Strong detection**	**8/8**	**0.00024**	**Catalyzes the removal of the acetyl group at the SN-2 position of platelet-activating factor**
			**8/8**	**0.00354**	
			7/8	0.011	
			6/8	0.027	
			4/8	0.051	
**Annexin A5**	**NM_001154**	**Strong detection**	**8/8**	**0.0012**	**Forms a two-dimensional protective shield, covering exposed potentially thrombogenic cell surfaces**
**Tissue factor pathway inhibitor**	**J03225.1**	**Strong detection**	**6/8**	**0.004**	**Inhibits tissue factor**
			3/8	0.076	
**Protein C receptor, endothelial**	**34335271**	**Strong detection**	**8/8**	**0.0044**	**N-glycosylated type I membrane protein that enhances the activation of protein C**
Thrombomodulin	40288292	Moderate detection	7/8	0.024	Receptor that binds thrombin activating protein C and degrades clotting factors Va and VIIIa
Protein S (alpha)	NM_000313	Moderate detection	7/8	0.024	A cofactor to anticoagulant activated protein C
Ectonucleoside triphosphate diphosphohydrolase 1	NM_001776	Moderate detection	1/8	0.081	Endothelial ecto-ADPase inhibiting platelet function via hydrolysis of released platelet ADP
**FIBRINOLYTIC FUNCTIONS**					
**Annexin A2**	**NM_004039**	**Strong detection**	**8/8**	**0.00024**	**Profibrinolytic co-receptor for tissue plasminogen activator and plasminogen on endothelial cells**
				**0.00024**	
				**0.00024**	
**Tetranectin (plasminogen-binding protein)**	**NM_003278**	**Strong detection**	**8/8**	**0.0022**	**Bind fibrin and coactivate plasminogen**
Plasminogen activator, urokinase	NM_002658	Moderate detection	7/8	0.024	Conversion of plasminogen to plasmin
			4/8	0.051	
Plasminogen activator, tissue	NM_000930	Moderate detection	7/8	0.031	Conversion of plasminogen to plasmin
Urokinase plasminogen activator receptor	X74039.1	Moderate detection	5/8	0.046	Binds uPA and permits activation of pro-enzyme by plasmin

Access # is NCBI accession number. Frequency is the number of biopsy samples showing significant expression out of a total number of 8 biopsies according to Affymetrix criteria. P-value is the median p-value of the Wilcoxon test for all 8 human biopsy samples. The gene was bolded and the p-value was underlined if it met the FDR criteria for being significantly detected. Multiple probe sets are available for some genes and are presented in the Table.

**Table 2:** Hemostatic gene expression in rat skeletal muscle tissue

**GENE**	**Access #**	**Expression**	**Frequency**	**P-value**	**Function**
**COAGULANT FUNCTIONS**					
**Tissue factor**	**U07619**	**Strong detection**	**8/8**	**0.0013**	**Initiates blood coagulation cascades, and it functions as the high-affinity receptor for the coagulation factor VII**
**Fibulin 5**	**NM_019153**	**Strong detection**	**8/8**	**0.0014**	**Forms complex with fibrinogen**
**Thrombin receptor**	**M81642**	**Strong detection**	**8/8**	**0.0066**	**Activated on surface of activated platelets by prothrombinase complex**
**Gamma-glutamyl carboxylase**	**AF065387**	**Strong detection**	**7/8**	**0.021**	**Carboxylation of coagulation factors VII**
P2Y purinoceptor	U22830	Moderate detection	5/8	0.031	Binds ADP and leads to platelet shape change
Coagulation factor IX	M26247	Moderate detection	2/8	0.058	Vit K dependent factor that activates factor X
Fibrinogen-gamma	J00735	Moderate detection	1/8	0.090	Fibrinogen is cleaved by thrombin to form fibrin
**ANTI-COAGULANT FUNCTIONS**					
**Annexin A5**	**D42137**	**Strong detection**	**8/8**	**0.00043**	**Forms a two-dimensional protective shield, covering exposed potentially thrombogenic cell surfaces**
Ectonucleoside triphosphate diphosphohydrolase1	NM_022587	Moderate detection	4/8	0.040	Endothelial ecto-ADPase inhibiting platelet function via hydrolysis of released platelet ADP
Protein S (alpha)	U06230	Moderate detection	2/8	0.044	A cofactor to anticoagulant activated protein C
Thrombomodulin	AF022742	Moderate detection	6/8	0.052	Receptor that binds thrombin activating protein C and degrades clotting factors Va and VIIIa
**FIBRINOLYTIC FUNCTIONS**					
**Plasminogen activator, tissue**	**M23697**	**Strong detection**	**8/8**	**0.00027**	**Conversion of plasminogen to plasmin**
**Annexin A2**	**L13039**	**Strong detection**	**8/8**	**0.00064**	**Profibrinolytic co-receptor for tissue plasminogen activator and plasminogen on endothelial cells**
**Plasminogen activator, urokinase**	**X63434**	**Strong detection**	**8/8**	**0.0026**	**Conversion of plasminogen to plasmin**

Access # is NCBI accession number. Frequency is the number of pools of muscle (n = 10 rats per pool) showing significant expression out of a total number of 8 pools of mRNA. P-value is the median p-value of a Wilcoxon test for all 8 muscle pools. The gene was bolded and the p-value was underlined if it met the FDR criteria for being significantly detected.

**Table 3:** LPP1 related gene expression in human skeletal muscle

**GENE**	**Access #**	**Expression**	**Frequency**	**P-value**	**Function**
**GENE SUPPRESSED BY PHYSICAL INACTIVITY**					
**LPP1**	**AB000888.1**	**Strong detection**	**8/8**	**0.004**	**Dephosphorylates LPA, S1P, PA, ceramide-1-P**
**GENES NOT AFFECTED BY PHYSICAL INACTIVITY**					
LPP2	AF047760.1	Not detected	0/8	0.47	Dephosphorylates LPA, S1P, PA, ceramide-1-P
**LPP3**	**NM_003713**	**Strong detection**	**8/8**	**0.0007**	Dephosphorylates LPA, S1P, PA, ceramide-1-P
			**8/8**	**0.0002**	
LPAP	AB031478.1	Moderate detection	2/8	0.068	Dephosphorylates LPA
SGPP1	NM_030791	Moderate detection	7/8	0.024	Dephosphorylates S1P
SGPL1	NM_003901.1	Not detected	0/8	0.90	Irreversible sphingosine 1-phosphate degradation
SPHK1	NM_021972.1	Not detected	0/8	0.19	Phosphorylates sphingosine
**AGPAT1**	U56417	**Strong detection**	**8/8**	**0.0029**	ER enzyme converting LPA to PA
			**8/8**	**0.0056**	
**AGPAT2**	U56418	Moderate detection	**8/8**	**0.028**	ER enzyme converting LPA to PA
**AGPAT3**	NM_020132	**Strong detection**	**8/8**	**0.002**	ER enzyme converting LPA to PA
AGPAT4	NM_020133.1	Not detected	0/8	0.25	Converts LPA to PA
**LYPLA1**	AF077198.1	**Strong detection**	**8/8**	**0.0002**	Hydrolyzes lysophosphatidylcholine to LPA
**LYPLA2**	NM_007260.1	**Strong detection**	**8/8**	**0.014**	Hydrolyzes lysophosphatidylcholine to LPA
EDG2	45580697	Moderate detection	4/8	0.068	LPA receptor
EDG3	NM_005226	Not detected	0/8	0.43	LPA receptor
EDG4	NM_004720.3	Not detected	0/8	0.63	LPA receptor
EDG7	NM_012152.1	Not detected	0/8	0.33	LPA receptor
EDG8	AF317676	Not detected	0/8	0.97	S-1-P receptor
P2Y9	NM_005296.1	Not detected	0/8	0.63	LPA receptor, platelet
ROCK2	D87931	**Strong detection**	7/8	0.010	LPA activated kinase

Access # is NCBI accession number. Frequency is the number of biopsy samples showing significant expression out of a total number of 8 biopsies. P-value is the median p-value of a Wilcoxon test for all 8 human biopsy samples. The gene was bolded and the p-value was underlined if it met the FDR criteria for being significantly detected. Multiple probe sets are available for some genes and are presented in the Table. ER, endoplasmic reticulum; LPA, lysophosphatidic acid; S1P, sphingosine-1-phosphate; PA, phosphatidic acid LPA, lysophosphatidic acid; S1P, sphingosine-1-phosphate; PA, phosphatidic acid; AGPAT, 1-acylglycerol-3-phosphate acyltransferase; EDG, endothelial differentiation gene; LPAP, LPA phosphatase; LPP, lipid phosphate phosphatase; LYPLA, lysophospholipase; ROCK2, Rho-associated, coiled-coil containing protein kinase 2; SGPL1, sphingosine-1-phosphate lyase 1; SGPP1, sphingosine-1-phosphatase; SPHK1, sphingosine kinase 1.

#### Differential expression

Several conservative and reliable criteria to define differential expression were utilized as previously described for rat tissues [16]. Multiple filters were used including a present call (p ≤ 0.04), a p-value ≤ false discovery rate (FDR) criteria and a magnitude of change ≥ 1.5-fold in the determination of differential expression. The use of such criteria has been shown to reduce both technical and biological variation. Normalization between microarrays is performed by the Affymetrix Microarray Suite 5.0 and normalizes the average intensity of both the treatment and control arrays. Unlike the desirable repeated measures experimental design in the human study, a cross-sectional study design was performed in rats (because of the inability to obtain serial biopsies from each rat). Thus, pooling of RNA for the microarrays from a large number of animals was used to minimize experimental error. For example, approximately 11 times more genes had a CV > 30% in single samples than in pooled samples (n = 8-10 per pool) [16]. For the acute rat study, each of the 3 hindlimb unloaded and the 2 treadmill exercised RNA pools was compared against each of the 3 control pools which resulted in 9 (3 x 3) comparisons for unloading and 6 (3 x 2) comparisons for treadmill exercise. For the chronic study, the two hindlimb unloaded pools were compared against the two control pools which resulted in 4 (2 x 2) comparisons. The average p-value from the Wilcoxon sign rank test of all these comparisons (either 9, 6, or 4 comparisons) was then ranked and compared against the FDR thresholds to determine differential expression [18]. In addition, only hemostatic related genes that were greater than 1.5-fold magnitude different between treatment and control for all the possible comparisons were considered as differentially expressed. For the human microarray analyses, only hemostatic related genes that were significantly expressed, greater than 1.5-fold different in each of the participants, and had a p-value from a paired t-test below 0.05 were considered as differentially expressed.

#### LPP1 PCR

Paired t-tests were used to compare the PCR derived expression of LPP1 during sitting with normal standing/ambulatory activity in humans. A p < 0.05 was considered statistically significant. All data is presented as means ± SEM.

## Results

### Expression of hemostasis genes in skeletal muscle

Genes were identified as being involved in hemostasis by searching PubMed and the publicly available Affymetrix NetAffyx website for key words related to hemostasis such as: coagulation, clotting, fibrinolysis, thrombosis, platelet aggregation, DVT, and hemostasis. First, we began this investigation in a large number of rats and the findings justified initiating a translational study in which human biopsies were obtained and extensively analyzed (44,792 probe sets). Genes involved in coagulation, anti-coagulation, and fibrinolysis are listed in Tables 1 (human) and 2 (rat). The absence and presence of expression for the set of genes related to LPP1 signaling are listed in Table 3. For some genes these arrays contained multiple sets of probes. Each single probe set for the human microarray contained 11 different mRNA target sequences of 25 nucleotides, and the results for both the better and the lower affinity probe sets are displayed in the Tables. This expands on the few hemostatic genes which have previously been reported to be expressed in skeletal muscle tissue.

### LPP1 expression is decreased during physical inactivity in rats

**Figure 1:**
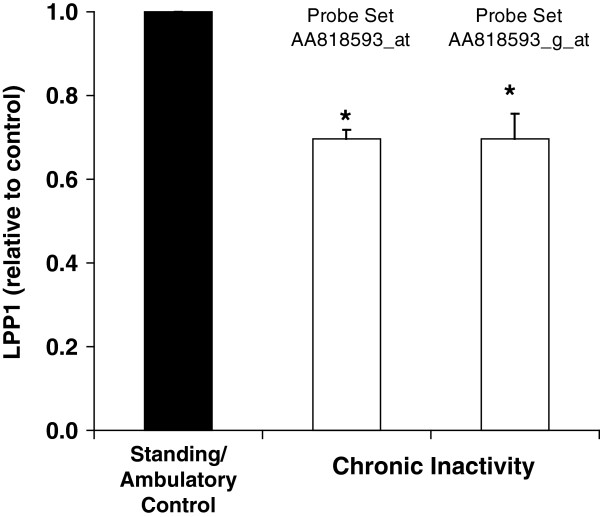
**Suppression of LPP1 gene expression in rat soleus caused by chronic physical inactivity.** Rats were prevented from standing for 10 hrs/d for 11 consecutive days. Results for two independent sets of probes for LPP1 on the rat U34A microarray are presented. Probe set nomenclature is from Affymetrix.com. Results are expressed as mean ± SE. † Inactivity vs. Ambulatory Control, p < 0.05.

**Figure 2:**
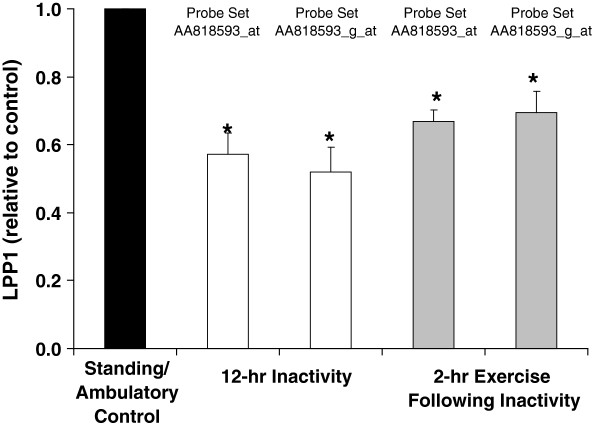
**Rapid suppression of LPP1 expression in rat soleus caused by 12 hours of physical inactivity is not restored by treadmill exercise 4 hours after beginning exercise.** Results are expressed relative to ambulatory control rat values. Results for two sets of probes on the U34A microarray are presented. Probe set nomenclature is from Affymetrix.com. Results are expressed as mean ± SE. * vs. Ambulatory Control, p < 0.05.

In order to identify genes potentially governing hemostasis that are most robustly affected by physical inactivity (not standing/ambulating) and non-exercise physical activity, we performed a microarray analysis where we looked for genes with hemostatic functions in rats. A single gene was differentially expressed in the acute study (LPP1) and, similarly, a gene was differentially expressed in a chronic study (Figure 1) (also LPP1). Interestingly, even exercise on a treadmill (30 minutes per hour) for 4 hours immediately following inactivity was not sufficient to rapidly restore LPP1 gene expression (Figure 2).

### LPP1 expression is decreased during physical inactivity in humans

**Figure 3:**
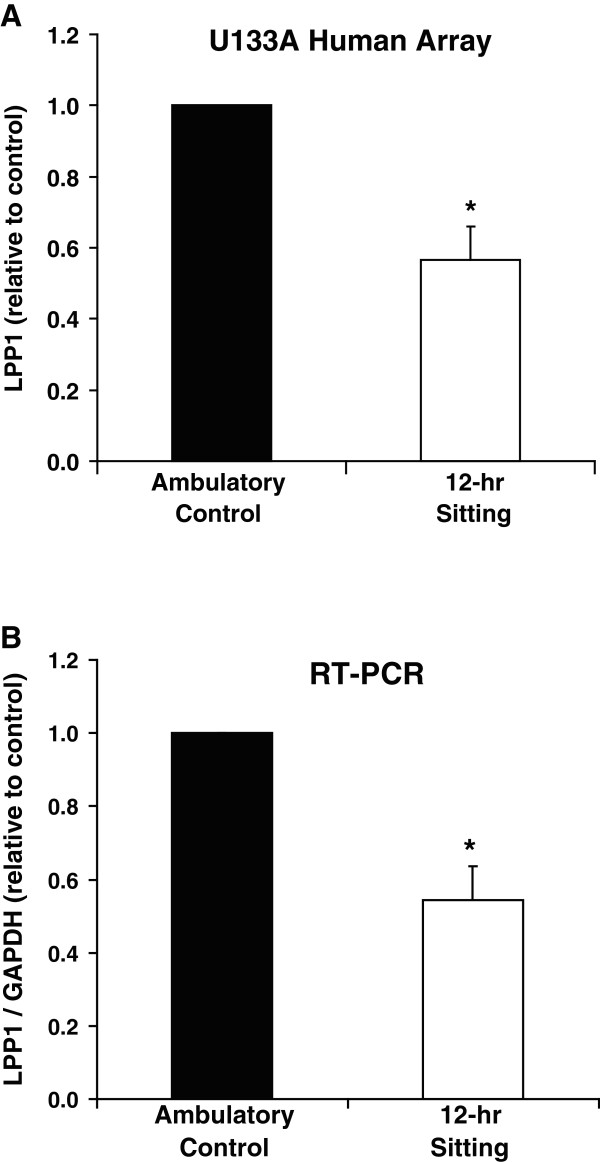
**Suppression of LPP1 mRNA expression in human skeletal muscle caused by 12 hours of physical inactivity.** Results are presented from (**A**) U133A human microarray data and (**B**) reverse transcriptase PCR. GAPDH mRNA expression was not affected by sitting. Results are expressed as mean ± SE. * vs. Ambulatory Control, p < 0.05.

Similar to the findings in the rat studies, microarrays revealed that 12 hours of physical inactivity (sitting) lowered LPP1 gene expression to 57 ± 9% of standing/ambulatory control levels (p < 0.05) (Figure 3A) in a repeated measures design (where each subject serves as their own control for comparison to sitting).

In accord with the microarray results, PCR results also revealed that sitting for 12 hrs reduced LPP1 gene expression (Figure 3B). LPP1 decreased on average to 55 ± 6% of the standing/ambulatory control values (p < 0.05).

### Case study

**Figure 4:**
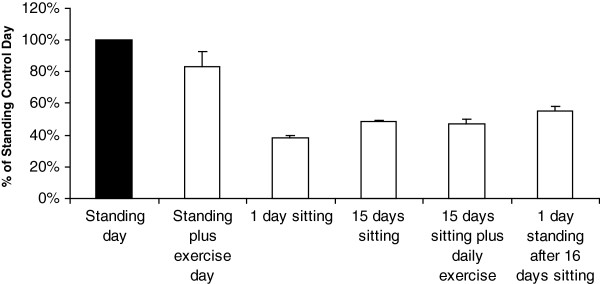
**Case study on the effect of several levels of physical activity on LPP1 expression in deep human skeletal muscle.** A healthy male subject (25 yrs.) was the tissue donor for all of the repeated measures treatments. The standing/ambulatory control day was normal to low-intensity puttering for 12 hours before the muscle biopsy. During the exercise day, the subject performed aerobic exercise (cycle ergometry and treadmill walking) in addition to normal spontaneous standing/ambulatory activity. For chronic inactivity, the subject sat in a wheelchair for 15 days and exercised one leg daily (but without adding back the natural standing and other low-intensity physical activity), while the other leg never received exercise. After 16 days of sitting, the subject returned to daily standing/ambulatory activity for 12 hours immediately preceding a final biopsy. Diet was controlled the day of and the day before all muscle biopsies which were all taken at the same time of day. Error bars are shown to indicate the variability (SD) between repeated biopsies on the same day.

In the follow-up study to repeat these measurements over a more extensive time course, the decrease in LPP1 expression was complete within the first day (41% of control, 3^rd^ bar in Figure 4) and was not decreased further after 15 days (49% of control, 4^th^ bar) of sitting in a wheelchair. During the chronic inactivity, this person exercised the left leg for one hour per day with cycle ergometry while the right leg never exercised. This daily exercise (i.e., 1 hr of vigorous cycling including intervals and resistance training, but no standing/ambulating) did not attenuate the decrease in LPP1 expression (5^th^ bar, Figure 4). Therefore, the effect of too much sitting overwhelmed any putative effects of exercise on LPP1. Furthermore, exercise was also ineffective in increasing LPP1 expression a month before the chronic inactivity (2^nd^ bar, Figure 4). We expected that LPP1 expression would return to normal following 12 hours of standing and ambulation after 16 days of sitting; it did not (56 ± 3% of standing control) (6^th^ bar, Figure 4).

Overall, these results suggest that physical inactivity (sitting in humans) has a robust and rapid effect of lowering LPP1 expression in both rats and humans. Or practically stated, standing and ambulatory activity is sufficient to maintain LPP1 expression at the normal level, while exercise does not appear to have any effect on LPP1 expression.

### Other genes related to LPP1 signaling

**Figure 5:**
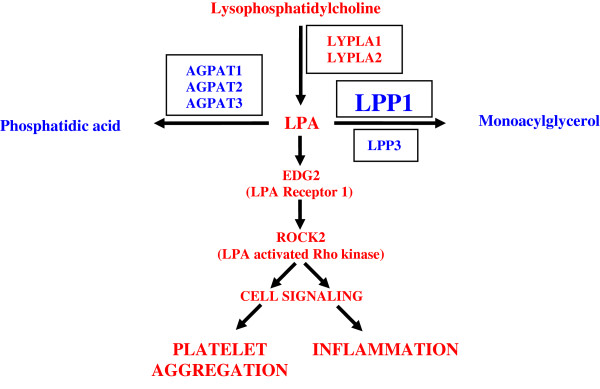
**Interaction of genes involved in lysophospholipid metabolism.** The novel finding is that skeletal muscle tissue expresses the genes regulating these pathways. The enzymes are in boxes and each is known to catalyze the reaction in the figure. The new data demonstrates that skeletal muscle tissue expresses a key LPA receptor (EDG2), and expresses a Rho kinase (ROCK2) well-known to be activated by LPA. In addition to platelet aggregation, ROCK2 has been associated with inflammation. The red pathway indicates a pathway to inflammation and the blue pathways would lead to an attenuation of LPA effects. Of these genes, only LPP1 is affected by physical inactivity. Abbreviations: LPC, lysophosphatidylcholine; LPA, lysophosphatidic acid; AGPAT, 1-acylglycerol-3-phosphate acyltransferase; EDG2, endothelial differentiation gene 2; LPP, lipid phosphate phosphatase; LYPLA, lysophospholipase; ROCK2, Rho-associated, coiled-coil containing protein kinase 2.

In addition to LPP1, there are several other lysophospholipid regulating genes expressed in skeletal muscle (Table 3 and Figure 5). Another isoform of LPP1, lipid phosphate phosphatase-3 (LPP3/PAP2B) was also significantly expressed above background (p < 0.05), although not significantly affected by physical inactivity. Lysophosphatidic acid (LPA) is putatively one of the key lipids involved in hemostasis, and is a substrate for LPP1. Two enzymes involved in LPA synthesis, lysophospholipase I (LYPLA1) and lysophospholipase II (LYPLA2), were significantly expressed in tandem with other genes in this pathway (p < 0.05). Three enzymes responsible for the transformation of LPA to phosphatidic acid (an initial step in triglyceride synthesis), lysophosphatidic acid acyltransferase-alpha (AGPAT1), lysophosphatidic acid acyltransferase-beta (AGPAT2), and lysophosphatidic acid acyltransferase-delta (AGPAT3) were significantly expressed. Of the LPA receptors (EDG2, EDG3, EDG4, EDG7) and the S-1-P receptor (EDG8), only EDG2 ( [19] LPA1 receptor) was moderately expressed in human skeletal muscle. The current understanding of the relationship between these genes is schematically represented in Figure 5.

## Discussion

This is the first study to comprehensively identify hemostasis related genes in both rodent and human skeletal muscle with microarray technology. We confirm the presence of a couple of previously reported genes (tPA and tetranectin) and extend this list to at least another 15 transcripts significantly expressed above background in skeletal muscle that have known roles in coagulation and fibrinolysis. Furthermore, we identified a potentially novel candidate gene, LPP1, that was sensitive to physical inactivity in both rat and human skeletal muscle, explaining why physical inactivity (too much prolonged sitting) may increase the risk for thrombosis. Because physical inactivity (e.g., sitting) is associated with DVT [4-6,11] and changes in the contractile state of muscle, we first raised the hypothesis in 2007 [3] that a change in the expression of a key gene(s) in deep skeletal muscle tissue would be evident and it could be a distinct response to the physiology of inactivity (sitting too much) apart from recommended exercise. LPP1, with a proposed antagonistic role in platelet aggregation [20] and inflammation [21], was significantly decreased in both the human and rat studies of muscular inactivity. LPP1 was decreased with both acute and chronic reductions in normal daily contractile activity but was not impacted by exercise.

We used Affymetrix microarrays with probes for over 44,000 human transcripts. From this search we identified 23 transcripts from genes with functions related to hemostasis that were at least moderately expressed according to Affymetrix standards in human muscle (Table 1). Previously, the protein for one of these (tPA) was reported to be detectable in human muscle biopsies [12,13]. Importantly, the more definitive detection of tPA (and tetranectin) at the mRNA level in human muscle [13] is consistent with our findings that one of the sources for these and other hemostatic factors could be within the muscle tissue itself, rather than indirect accumulation of the protein from the blood. We have here confirmed and extended these observations to other genes involved in fibrinolysis (uPA and uPA receptor, annexin A2), and also to genes involved in blood coagulation (i.e., Factors VII and VIII, thrombin receptor, and vWF). Furthermore, the mRNA for two enzymes, gamma-glutamyl carboxylase and vitamin K epoxide reductase, necessary for the synthesis of coagulation factors, were expressed in skeletal muscle. Despite some differences in muscle fiber type between species, all of the genes with anti-coagulant and fibrinolytic functions detected in the rat muscle were also expressed in the human muscle (i.e., annexin A5, ectonucleoside triphosphate diphosphohydrolase 1, protein S (alpha), thrombomodulin, tPA, annexin A2, uPA). This agrees with a general tenet in muscle physiology that skeletal muscle is relatively conserved between species compared to other tissues. This observation of similar anti-coagulant and fibronolytic genes being expressed in the skeletal muscle of both rats and humans would support the use of rodent models in translational studies of muscle and hemostasis.

**Figure 6:**
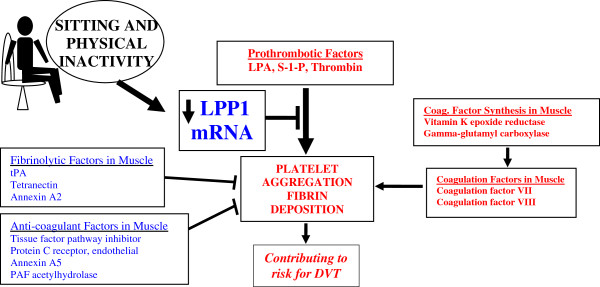
**Relationship between hemostatic gene function, physical inactivity, LPP1 and risk of deep venous thrombosis.** Human skeletal muscle expresses distinct groups of genes involved in hemostasis (fibrinolysis, anti-coagulation, and coagulation factors, and enzymes involved in the synthesis of these factors). Of all these genes expressed in skeletal muscle, only LPP1 expression was affected by physical inactivity (i.e., sitting in humans). Sitting in humans and the removal of standing in rats suppressed LPP1 expression in skeletal muscle tissue. Prior studies have indicated that DVT is a complex disorder and caused by many interacting factors. We propose the novel hypothesis that the local gene expression deep in skeletal muscle could be a contributing factor. Many candidate genes known to regulate platelet aggregation and fibrin deposition are presently reported to be expressed in muscle tissues (Tables 1 and 2) and are presented here as four distinct groups. While some of those could be regulated post-transcriptionally, LPP1 mRNA was significantly decreased by physical inactivity. Prior published work indicates that LPP1 attenuates platelet aggregation, fibrin deposition, and inflammation. Ordinary non-exercise movements and standing, unlike the less frequent and higher intensity type of exercise more commonly associated with a boost in muscular strength or cardiovascular fitness, prevent the decrease in LPP1 expression. Items in red would favor a prothrombotic state while those in blue would be anti-thrombotic.

The new knowledge that LPP1 is expressed in leg tissue differentially by physical activity patterns is significant because it provides the first study we are aware of on the physiological regulation of this gene. LPP1 mRNA encodes a 32 kDa transmembrane ecto-enzyme responsible for the degradation of the extracellular bioactive phospholipids by dephosphorylation. Lysophosphatidic acid (LPA), sphingosine-1-phosphate, ceramide-1-phosphate, and phosphatidic acid are all substrates for LPP1. LPA is arguably the most important of these lipids because it has the highest affinity for LPP1, and even more importantly, because LPA has recently emerged as a potent stimulator of platelet aggregation [20,22-25], platelet monocyte aggregation [20], tissue factor expression [26], fibronectin matrix [27], and inflammation [21,28,29]. Similarly, sphingosine-1-phosphate has been shown to stimulate platelets to bind and assemble fibronectin [27], stimulate tissue factor expression [31], and inflammation [28]. Both LPA and sphingosine-1-phosphate are released from activated platelets and are thought to promote a positive feedback on platelet aggregation. Haseruck et al. [20] argued that the expression of LPP1 on the endothelium of tissues (such as the endothelium in muscle tissue) would attenuate local accumulation of LPA and thus limit platelet activation [20]. In cell culture experiments where LPP1 expression has been manipulated, LPP1 has been shown to be important for the attenuation of several atherothrombogenic and malignant processes induced by LPA, including platelet aggregation [25], the release of the proinflammatory cytokine IL-8 [22], and ovarian cancer cell proliferation [32]. There are several lines of evidence that LPP1 can also attenuate the signaling of other factors including the prothrombotic and proinflammatory cytokine TNFα [21] and thrombin [19]. Therefore, if the protein is regulated by its gene expression, LPP1 would be a novel candidate for why physical inactivity is a risk factor for DVT or other types of thrombosis (Figure 6).

Other genes involved in LPA metabolism were detected in skeletal muscle (Table 3 and Figure 5). An isoform of LPP1, LPP3, is significantly expressed in human skeletal muscle and is similar to LPP1 in that it degrades extracellular LPA and has been shown to attenuate LPA induced signaling in ovarian malignant epithelial cell cultures [33]. In contrast to LPP1’s location on the apical side of endothelial cells, LPP3 is thought to be located on the basolateral membrane of endothelial cells [34]. The enzyme responsible for the conversion of lysophosphatidylcholine to LPA, lysophospholipase I, was strongly detected in both human and rat skeletal muscle and, therefore, may play a local role in LPA production.

### Limitations

Although we present robust data showing that physical inactivity can reduce LPP1 transcript level, we did not determine if this was due to a reduction in transcription (possibly secondary to epigenetic changes to chromatin or DNA) or a reduction in mRNA stability or both. In addition, we report only on the mRNA level of this gene and not on the protein as there are not good antibodies yet available for this transmembrane protein. As we tested only lean and apparently healthy rats and humans we cannot determine that LPP1 expression would be responsive to physical inactivity in a more diseased population.

## Conclusions

This study raises the novel hypothesis that muscle disuse may contribute to hemostatic disorders not simply because of factors related to reduced blood flow, but also potentially because of the diverse repertoire of hemostatic genes expressed locally in muscles, such as LPP1. These findings highlight the importance of the concept of inactivity physiology where processes engaged by physical inactivity (not standing or ambulating) are not necessarily the opposite of exercise [3]. This might explain why exercise in the present study was ineffective at counteracting the effects of physical inactivity (Figures 2 and 4). There is little doubt that DVT is not caused by a lack of participation in exercise training programs, but it appears that DVT is caused in part by the absence of the frequent contractions normally associated with low-intensity movements throughout the day. The preliminary findings of this study need to be investigated at multiple scientific levels to understand the putative role of LPP1 and lysophospholipid metabolism in risk for thrombosis associated with a lack of skeletal muscle contractile activity. Finally, these findings of a number of hemostatic genes expressed in skeletal muscle may emphasize the potential role of skeletal muscle in health and disease as more than simply contractile machinery.

## Competing interests

The authors declare that they have no competing interests.

## Authors’ contributions

TWZ and MTH were responsible for the research design, data collection, biochemical analyses, data analyses and manuscript writing. Both authors approved the final submission.
